# Crosstalk between dendritic cells and regulatory T cells: Protective effect and therapeutic potential in multiple sclerosis

**DOI:** 10.3389/fimmu.2022.970508

**Published:** 2022-09-13

**Authors:** Ruoyu Li, Hui Li, Xiaoyan Yang, Huiru Hu, Peidong Liu, Hongbo Liu

**Affiliations:** ^1^ Department of Neurology, The First Affiliated Hospital of Zhengzhou University, Zhengzhou, China; ^2^ Department of Neurosurgery, First Affiliated Hospital of Zhengzhou University, Zhengzhou, China; ^3^ Translational Medicine Center, First Affiliated Hospital of Zhengzhou University, Zhengzhou, China

**Keywords:** dendritic cell, regulatory T cell (Treg), immune tolerance, multiple sclerosis, experimental autoimmune encephalomyelitis

## Abstract

Multiple sclerosis (MS) is a chronic inflammatory disease of the central nervous system related to autoimmunity and is characterized by demyelination, neuroinflammation, and neurodegeneration. Cell therapies mediated by dendritic cells (DCs) and regulatory T cells (Tregs) have gradually become accumulating focusing in MS, and the protective crosstalk mechanisms between DCs and Tregs provide the basis for the efficacy of treatment regimens. In MS and its animal model experimental autoimmune encephalomyelitis, DCs communicate with Tregs to form immune synapses and complete a variety of complex interactions to counteract the unbalanced immune tolerance. Through different co-stimulatory/inhibitory molecules, cytokines, and metabolic enzymes, DCs regulate the proliferation, differentiation and function of Tregs. On the other hand, Tregs inhibit the mature state and antigen presentation ability of DCs, ultimately improving immune tolerance. In this review, we summarized the pivotal immune targets in the interaction between DCs and Tregs, and elucidated the protective mechanisms of DC-Treg cell crosstalk in MS, finally interpreted the complex cell interplay in the manner of inhibitory feedback loops to explore novel therapeutic directions for MS.

## Introduction

**Figure 1:**
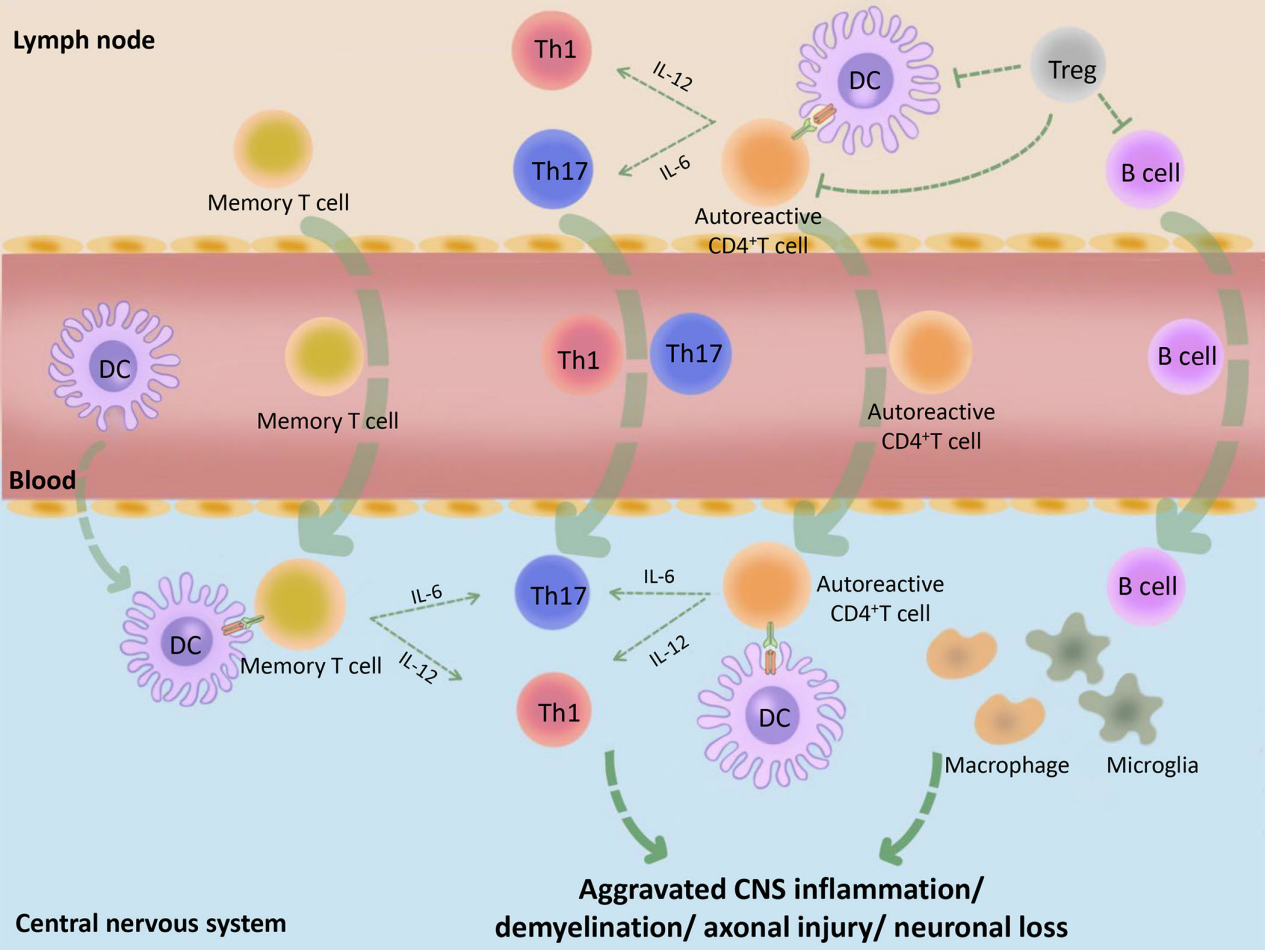
Basic immunopathological process of MS. Initially, the peripheral tolerance of MS patients is disordered (for example, the inhibitory function of Tregs is decreased or the Teffs are not sensitive to the inhibitory effect of Tregs) and the autoantigens, such as MBP, MOG and PLP, are incorrectly recognized as pathogens by DCs. These DCs combine with CD4^+^ autoreactive T cells, which cause peripheral immune response. The activated immune cells make entering into the peripheral blood, then trafficking across the blood–CNS barrier. Subsequently, activated DCs in the CNS combine and reactivate with memory T cells or CD4^+^ autoreactive T cells and promote their differentiation into Teffs, such as Th1 and Th17, followed by causing CNS inflammation. At the same time, B cells, macrophages, microglia and many other immune cells are also activated by the inflammatory context, followed by causing demyelination, axonal damage, and more serious CNS inflammation. MBP, myelin basic protein; PLP, proteolipid protein; MOG, myelin oligodendrocyte glycoprotein.

Multiple sclerosis (MS) is an immune-mediated disease characterized by inflammatory demyelinating lesions in the central nervous system (CNS), which causes the youngs non-traumatic disability. The course of MS is characterized by recurrence and remission and MS is thus divided into three main clinical types: relapsing-remitting MS (RRMS), primary progressive MS (PPMS), and secondary progressive MS (SPMS). Existing studies have shown that MS is mainly mediated by myelin-specific autoreactive CD4^+^T cells, whereas dendritic cells (DCs), regulatory T cells (Tregs), and other immune cells play auxiliary roles. When the peripheral immune tolerance is disordered due to Treg cell defects and/or the resistance of effector cells to Tregs, autoreactive CD4^+^ T cells in the lymph nodes are activated and become aggressive effector cells, followed by entering into the peripheral blood and trafficking to the brain. Besides, DCs, memory T cells, and other immune cells, also cross the blood-brain barrier into the CNS under the action of chemokines (1, 2). Subsequently, activated DCs reactivate autoreactive CD4^+^ T cells and memory T cells, and prompt their polarization into Teffs, such as T helper (Th) type 1 and Th17 *via* the different cytokine environment, followed by causing an inflammatory cascade reaction (3, 4). In addition, the activated T cells mediate the activation of macrophages as well as microglia and assist B cells to produce antibodies. When patients with MS enter the chronic phase, immune cell infiltration appears in the CNS and activated DCs, macrophages, and microglia further damage the myelin sheaths, axons, and neurons, which leads to the progression of MS (3) (**Figure 1**). Therefore, in individuals with MS, it appears that immune dysregulation originates from DCs with the activation phenotype that initiates adaptive immune responses (3). In addition, Tregs, as immune supervisors, can inhibit the inflammatory immune response mediated by activated DCs, pathogenic B cells, and T cells (5). Besides, a small number of potential tolerogenic DCs play a limited protective role in MS, such as inducing Treg cell generation and effector T cell (Teff) anergy. Consequently, the occurrence of MS might be related to the limited DC ability to induce normal Tregs or the insufficient monitoring of activated DCs by Tregs.

DC-Treg cell crosstalk has also been shown to affect animal model of MS-experimental autoimmune encephalomyelitis (EAE). For example, the number of CNS-infiltrating Tregs was decreased in DC deficient EAE mice (6). Similarly, during the initiation phase of activation in myelin-specific T cells, conditional ablation of CD11c^+^ DCs selectively suspended the generation of induced Tregs (iTregs) and resulted in the aggravation of EAE (6, 7). In turn, Tregs also shaped the tolerance of plasmacytoid DCs (pDCs) in a steady state, which was conducive to maintaining the regulatory ability of Tregs during EAE (8). A basic study has shown that Tregs can reduce the stable contact between DCs and naive T cells and interfere with the initiation of CD4^+^ T cells in the early immune response (9). Moreover, activated antigen-specific Tregs can also inhibit DC antigen presentation *via* the bystander effect (10). Detailed examinations of the crosstalk mechanisms of DC-Treg revealed that DCs influenced the proliferation, differentiation, and function of Tregs by reducing the expression of co-stimulatory molecules on the surface of the cell membrane, as well as by enhancing the expression of co-inhibitory molecules, metabolic enzymes, and the secretion of anti-inflammatory cytokines. Besides, Tregs can limit DC maturation and antigen presentation ability by secreting anti-inflammatory cytokines and boosting the formation of co-inhibitory signaling pathways. These processes can interfere with the metabolic pathways of DCs and even directly destroy DCs *via* cytolysis (11, 12). Various cell therapies for MS make full use of the DC-Treg cell crosstalk mechanism to achieve a therapeutic effect; these include tolerogenic dendritic cell (tolDC) therapy and regulatory T cell therapy (13). Tolerogenic dendritic cells mainly maintain highly specific immune tolerance by inducing the anergy and deletion of Teffs, and producing myelin-specific Tregs (13, 14). Similarly, Tregs can also establish antigen-specific immune tolerance by eliminating over-activated DCs and Teffs, and inducing the generation of tolDCs (10, 13). Although both cell therapies have broad application prospects in MS, there remain numerous barriers to the translation of knowledge into practical use. Thus, the protective DC-Treg cell crosstalk mechanism is crucial to inducing the immune tolerance in MS/EAE, and further in-depth studies will be instrumental to translate the protective mechanism into cell therapy.

By analyzing the co-stimulatory molecules, cytokines, and metabolic enzymes related to the DC-Treg cell interplay, this review comprehensively elucidates the protective effect of the DC-Treg cell crosstalk mechanism in MS/EAE and attempts to interpret the complex cell interaction *via* novel inhibitory feedback loops, with the further goal of exploring new research directions for protective autoimmune mechanisms and therapeutic schedules for MS.

## Classification of DCs and Tregs

DCs are the most powerful antigen-presenting cells (APCs), which exist in almost all human peripheral tissues and link innate and adaptive immune responses. DCs can be divided into conventional DCs (cDCs), pDCs and monocyte-derived DCs (MoDCs) (15–18). And cDCs can be further divided into the type 1 (cDC1) and type 2 (cDC2) subsets (15, 16, 19–21)(**Table 1**). Based on the characteristics of cellular immune tolerance, DCs can also be divided into immunogenic DCs and tolDCs (22). At present, there is no specific surface markers for tolDCs, although it can be identified by several characteristics:1) low expression of co-stimulatory (CD83 and CD80/86) and major histocompatibility complex class II (MHC-II) molecules and pro-inflammatory cytokines (interleukin [IL]-6, IL-12); 2) high expression of co-inhibitory molecules (programmed cell death ligand 1 [PD-L1]), anti-inflammatory cytokines (IL-10, IL-35, and transforming growth factor [TGF]-β), and indoleamine 2,3-dioxygenase (IDO) (23). In the steady state, most DCs maintain in an immature condition, and the types of antigens (i.e., pathogen or self-antigen) encountered with DC (including immature DC and mature DC) usually determine whether it plays an immunogenic or tolerogenic role (20, 24). However, in MS, most DCs are regarded as immunogenicity because the immune system mistakenly recognizes the self-antigens (i.e., myelin basic protein [MBP], proteolipid protein [PLP] and myelin oligodendrocyte glycoprotein [MOG]) as pathogens. Intriguingly, in MS, several rare DC subsets expressing CD8, CD103, dendritic cell endocytic receptor (DEC205), B- and T-lymphocyte attenuator (BTLA) or secreting interleukin (IL)-10, IL-27, IL-35 might become the potential tolDC subsets that induce Tregs. Therefore, DCs have two main functions in MS: one is playing a role in antigen presentation, activating T cells, and initiating immune responses; the other is interacting with T cells, shaping the differentiation/development/function of T cells, and inducing immune tolerance (22). The antigen presentation ability of DCs, namely immunogenicity, can be compensated by macrophages and B cells; however, the tolerance function of DCs appears irreplaceable (6).

**Table 1:** Human dendritic cell subsets related to multiple sclerosis.

	Function introduction		Marker	Ref.
cDC	cDCs initiate from the myeloid progenitors. In the process of cDC maturation, the ability to capture environmental- and cell-associated antigens, and initiate adaptive immune responses will be enhanced, while the ability of phagocytosis and elimination of pathogens will be relatively weakened. In the steady state (mouse), the marker combination, CD11c and MHCII, can distinguish migratory DCs (CD11c^int^MHCII^hi^) from lymph node resident DCs (CD11c^hi^MHCII^int^) phenotypically. During inflammation (mouse), CD8α on LN-resident cDC1s and CD103 on migratory cDC1s can be their own special mark, while such a marker does not exist in resident and migratory cDC2s.	cDC1	CD141(BDCA3), CLEC9A, XCR1, CD205(DEC-205) *	(15, 16, 19)
		cDC2	CD1c (BDCA1), CD301 (CLEC10A, MGL), CD172a (SIRPα), DCIR2*	(21)
pDC	pDCs, which are morphologically similar to plasma cells that produce antibodies, initiate from the lymphoid progenitors. pDCs demonstrate strong activation in response to viral and bacterial infections, secreting massive amounts of type I-IFN and acquiring the ability to present foreign antigens. pDCs express low levels of MHC-II, co-stimulatory molecules and the integrin CD11c in the steady state. When they are not stimulated, pDCs show a tolerogenic potential.		CD303 (BDCA2), CD123, CD304, CD45RA	(15, 16)
MoDC	Monocyte-derived DCs (MoDCs), which also named as “inflammatory DCs”, arise from the myeloid progenitors, and play a complementary role to cDCs. Phenotypically, we can roughly distinguish cDCs and MoDCs with CD11b.		FCER1A, CD14, CD226, CD64, CD1a*, CD1b*, CD1c*	(17, 18)

BDCA3, blood DC antigen 3; CLEC9A, C-type lectin domain-containing 9A; XCR1, the chemokine XC receptor 1; MHC II, major histocompatibility complex class II; FCER1A, Fc epsilon receptor Ia. *: less important than the others.

Tregs are a classical type of inhibitory T cell with an immune regulation function, which are primarily involved in the formation of immune tolerance. Tregs can be divided into two subsets: thymus derived Tregs (tTregs) and peripheral derived Tregs (pTregs). The main phenotype of tTregs is CD4^+^ CD25^+^ CD127^−^ Foxp3^+^ Tregs, while pTregs mainly include CD4^+^ CD25^+^ Foxp3^−^ type 1 regulatory T cells (Tr1) that secrete a large amount of IL-10 and CD4^+^ CD25^low^ T helper type 3 cells (Th3), which produce significant amounts of TGF-β. In addition, there are many Treg subsets that have been named based on key surface molecules or transcription factors, such as CD8^+^ Tregs and FoxA1^+^ Tregs (25). The heterogeneity of Tregs makes their subsets complex, and we have summarized the main Treg cell subsets related to MS (25–32) (**Table 2**). Tregs not only interfere with the mature state and antigen presentation ability of DCs but also induce the generation of tolDCs. When DCs play an excessive role in antigen presentation, and attack both pathogenic cells and normal cells, Tregs act as a negative regulator of these overactivated DCs and restrict the autoimmune response.

**Table 2:** Human regulatory T cell subsets related to multiple sclerosis.

Subset	Marker	Distribution	Characteristics	Ref.
Thymus-derived Treg cell (tTreg)	CD4^+^CD25^+^Foxp3^+^	Thymus and peripheral tissues	They enter the Treg cell lineage as a consequence of intermediate or strong TCR signals during T cell maturation in the thymus.	(26)
Type 1 regulatory T cell (Tr1)	IL-10-producing CD4^+^ CD25^-/low^ Foxp3^-^	Peripheral tissues	They secrete predominantly IL-10 and have a suppressor function independent from Foxp3. Memory Tr1 cells also regard LAG-3 and CD49b as biomarkers.	(27)
T helper type 3 cell (Th3)	TGF-β-producing CD4^+^CD25^-/low^	Peripheral tissues	They secrete predominantly TGF-β. The suppressive ability of Th3 and Tr1 cells is contact-independent and is based mainly on cytokines such as TGF-β and IL-10.	(28)
Th1-like, Foxp3^+^ Treg cell	T helper type 1-like IFN-γ-secreting Foxp3^+^	Peripheral blood	They show reduced suppressive activity. Moreover, Tregs associated with MS were excessively inclined to acquire Th1-Treg phenotype.	(29)
T follicular regulatory cell (Tfr)	CXCR5^+^ PD-1^+^ BCL6^+^ Foxp3^+^ CD25^-^	Peripheral tissues	The traditional viewpoint is that Tfr cells are negative regulators of B cells and Tfh cells in germinal centers. However, the function of Tfr cells is multifaceted in fact, which may reflect the function of different subsets of Tfr cells.	(30, 31)
CD8^+^ Treg cell	Foxp3^+^CD8^+^ /CD8^+^CD28^-^	Peripheral tissues and thymus	Presenting in the thymus, tonsils, and peripheral blood of human, CD8^+^ Tregs can mitigate spontaneous germinal center reactions and various immune responses via suppressing Tfh cells and B cells.	(30, 32)
FoxA1^+^ Treg cell	CD4^+^FoxA1^+^CD47^+^ CD69^+^PD-L1^hi^ FoxP3^-^	CNS and peripheral blood	FoxA1^+^ Tregs develop primarily in the CNS and respond to autoimmune inflammation in a FoxA1- and PD-L1-dependent manner.	(25)

## Defects in DCs and Tregs in MS

A recent study indicated that monocyte-to-DC differentiation was a process potentially influenced by MS risk single nucleotide polymorphisms (SNPs) (33). Moreover, previous studies showed that MS patients had DC defects. For example, the percentage of circulating myeloid DCs was decreased in patients with SPMS and PPMS, whereas a large number of DCs were found in the CNS lesion and cerebrospinal fluid (CSF) (34). The process of DC exiting the CNS to maintain immunological tolerance was perturbed in MS patients, which might contribute to the abnormal distribution of DCs in MS patients (2). In the CSF, pDCs percentage was higher in untreated-relapsing MS patients than those of patients in remission, whereas this result was not observed in the peripheral blood (35). In contrast, the expression of CD83, CD80, and CD86 on the surface of circulating DCs in patients with PPMS was downregulated, whereas the circulating cDCs of RRMS and SPMS patients showed an activated phenotype, suggesting an imbalance between tolerogenicity and immunogenicity of DCs in MS patients (i.e., functional defects in DCs) (34, 36). In addition, the ability of pDCs to induce Tregs has been shown to be related to the occurrence of MS. Gene expression level of HSPA1A —which may influence the ability of pDCs to induce Tregs—was downregulated in MS patients and this probably resulted in alterations in the mature state and regulatory function of pDCs (37). Furthermore, the depletion of pDCs in healthy controls resulted in the reduction in CD4^+^ Foxp3^+^ Tregs, whereas the depletion of pDCs in MS patients did not influence the generation of Tregs (38). Taken together, these studies showed that DC deficiency was closely related to MS relapsing and remitting.

Treg cell defects also exist in MS patients. CD25, namely the IL-2 receptor α chain, is a pivotal surface marker of Tregs, and its SNPs are closely related to MS (39). Studies have demonstrated that Treg cell defects in MS patients were mainly reflected in cell quantity, subset changes, and dysfunction (40–43). For example, the percentage of Tregs in the peripheral blood of RRMS patients was significantly reduced and associated with the clinical severity of the disease (41). Moreover, a previous study has shown that the number of Tregs in the CSF, but not in the peripheral blood, was elevated in MS patients (42). In contrast, alterations in the proportion and dysfunction of Treg cell subsets were more marked in patients with MS (43). For example, the effector function of CD4^+^ CD25^hi^ Tregs in the peripheral blood was significantly downregulated in MS patients (44). Moreover, T helper type 1 (Th1)-like, interferon-γ (IFN-γ)-secreting Foxp3^+^ T cells were elevated in RRMS patients, while their inhibitory function were weakened (29). A recent study showed that, compared with healthy donors, MS patients had lower resting CD4^+^ CD25^+^ CD45RA^+^ CCR7^+^ Tregs and more activated CD4^+^ CD25^hi^ CD45RA^-^ Foxp3^hi^ Tregs even before treatment (40). Therefore, Treg cell deficiency was one of the main causes of the autoimmune response in MS patients.

## Bidirectional DC-Treg cell crosstalk in MS patients and EAE mice

**Figure 2:**
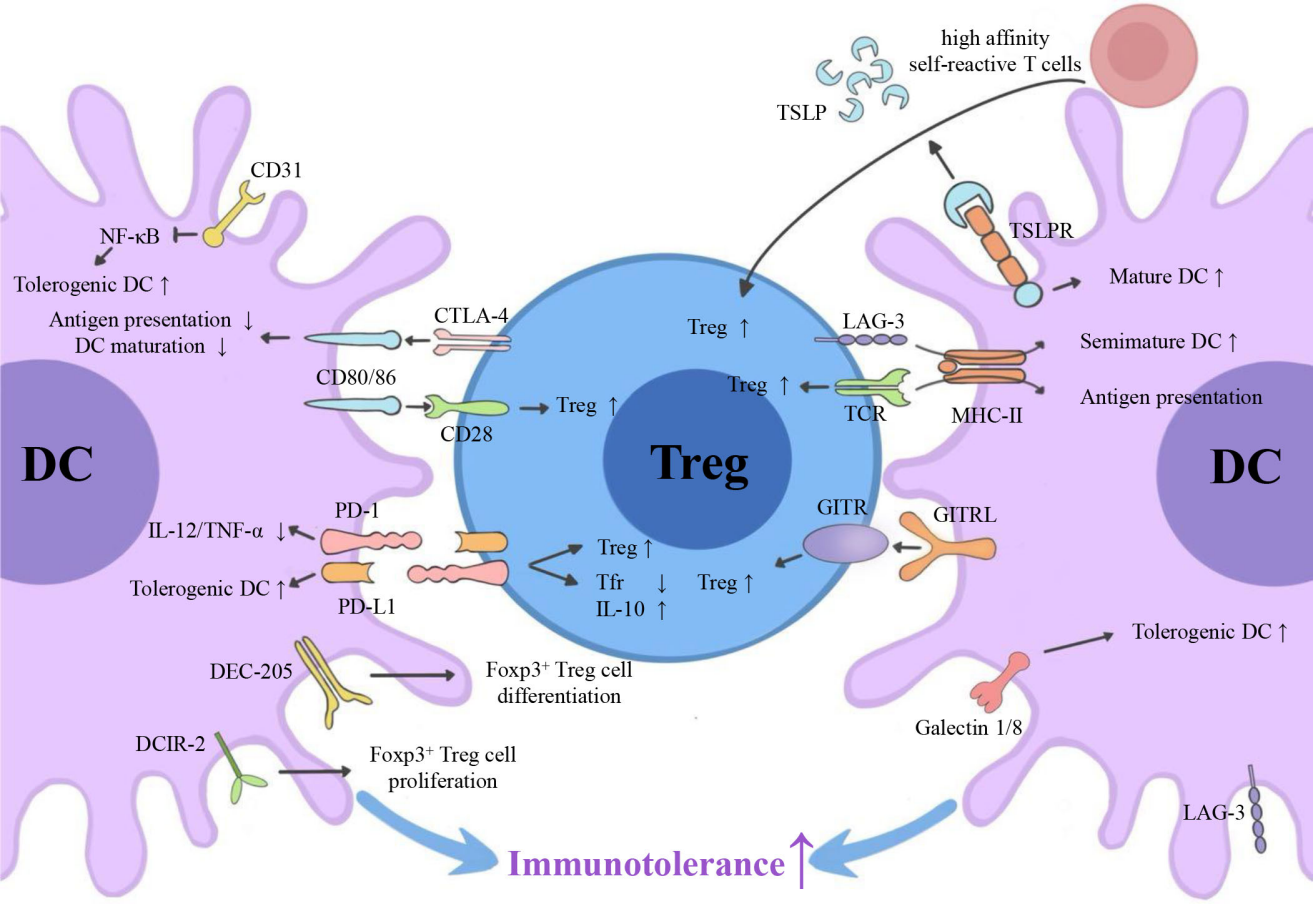
Surface molecules involved in DC-Treg cell crosstalk. *Via* the CD28/CTLA-4/B7 pathway, the PD-L1/PD-1 inhibitory feedback loop, and the potential LAG-3/TCR/MHC-II inhibitory feedback loop, DCs can affect the generation of Tregs, and Tregs can affect the tolerance of DCs. GITRL, TSLPR, DEC-205, DCIR-2, and CD31 are also key molecules for DC-dependent Treg cell generation. CTLA-4, cytotoxic T lymphocyte antigen 4; PD-L1, programmed cell death ligand 1; PD-1, programmed cell death receptor; LAG-3, lymphocyte activation gene 3; TCR, T cell receptor; MHC-II, major histocompatibility complex class II; GITR, glucocorticoid-induced tumor necrosis factor receptor; GITRL, glucocorticoid-induced tumor necrosis factor receptor ligand; TSLP, thymic stromal lymphopoietin; DCIR2, dendritic cell inhibitory receptor 2.

**Figure 3:**
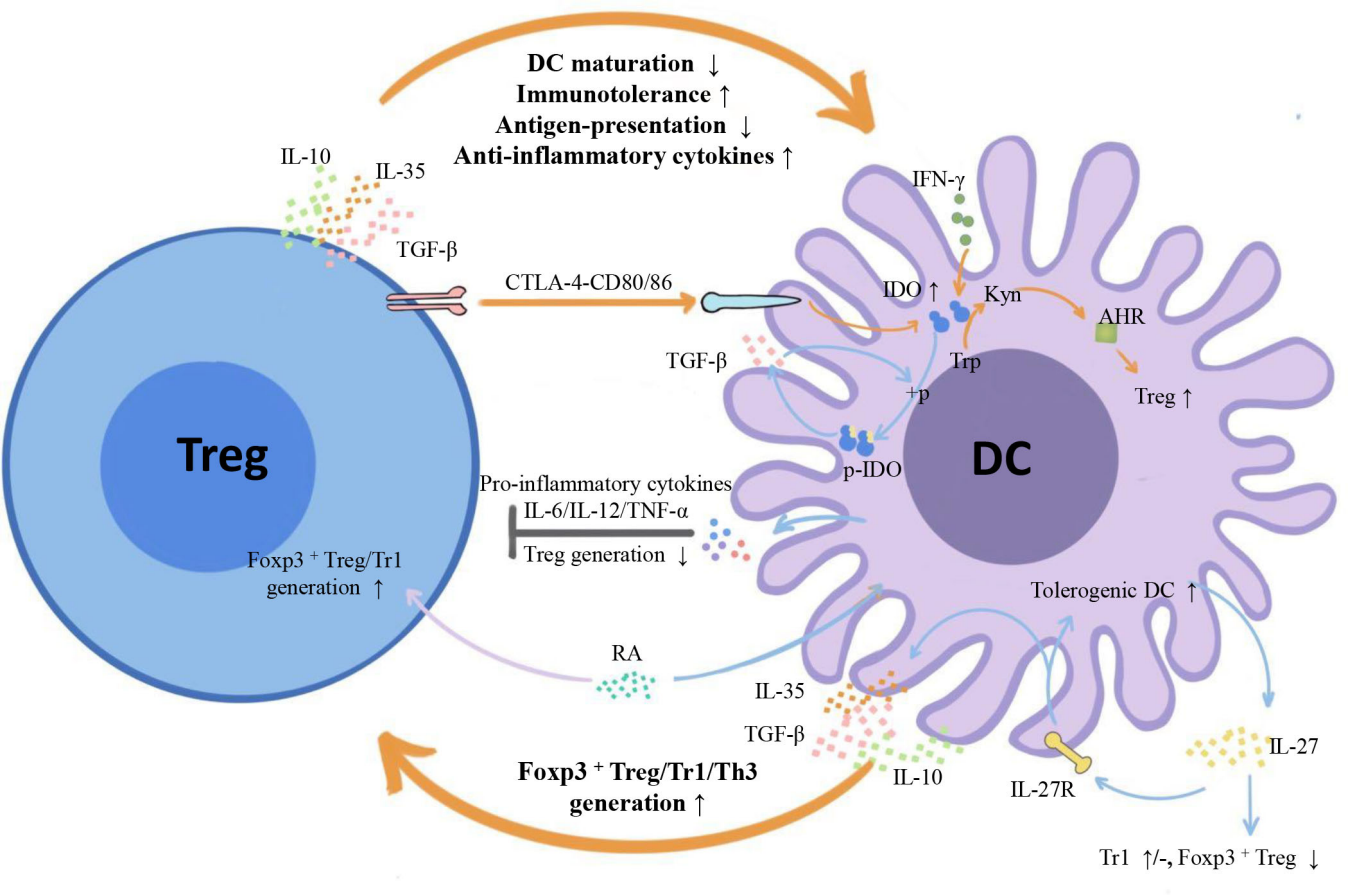
Inhibitory cytokines and metabolic enzymes involved in DC-Treg cell crosstalk. From the perspective of cytokines, both DCs and Tregs can secrete inhibitory cytokines IL-10/IL-35/TGF-β and form inhibitory feedback mechanisms: DCs promote the generation of different subtypes of Tregs, and Tregs inhibit the maturity and antigen presentation ability of DCs and improve their tolerance. From the perspective of metabolic enzymes, Tregs can induce IDO expression in DCs with the help of signals axes, such as CTLA-4-CD80/86, initiate the tryptophan metabolic pathway, trigger the generation of IDO-dependent Tregs, and form a negative feedback loop with IDO at the core. There is also unidirectional regulation of Tregs by DCs: DCs influence Treg generation by secreting IL-27, and IL-12/IL-6/TNF-α inhibits Treg cell proliferation. TGF-β, transforming growth factor-β; IFN- γ, Interferon- γ; IDO1, indoleamine 2,3-dioxygenase 1; + p, phosphorylation; P-IDO1, phosphorylated indoleamine 2,3-dioxygenase 1; Trp, tryptophan; Kyn, kynurenine; AHR, aromatic hydrocarbon receptor.

Emerging myeloid cell research has been focused primarily on the regulatory effect of DCs on Tregs. However, the regulatory effect of Tregs on DCs is also worth investigating. We summarize three inhibitory feedback loops to explain the protective crosstalk mechanism between DCs and Tregs in the context of MS/EAE: the CD28/cytotoxic T lymphocyte antigen (CTLA)-4/B7 pathway, the PD-L1/PD-1 inhibitory feedback loop, and the lymphocyte activation gene 3 (LAG-3)/T cell receptor (TCR)/MHC-II inhibitory feedback loop (**Figure 2**). We also highlight three inhibitory feedback mechanisms related to DC-Treg cell crosstalk that may be involved in MS/EAE, including IL-10, IL-35, and TGF-β (**Figure 3**).

### Surface molecules involved in DC-Treg cell crosstalk

#### Protean CD28/CTLA-4/B7pathway

CD28 and CTLA-4 (CD152) are members of the CD28 superfamily. They share common ligands, the B7 family molecule CD80/CD86 (B7-1/B7-2), on the surface of DCs. The CD28 molecules that are crucially expressed in most CD4^+^ T cells (including Tregs) promote T cell activation, proliferation, and survival (45). Besides, the expression of CTLA-4 is pivotal in Tregs and is upregulated on the surface of activated T cells. CTLA-4 has considerably higher avidity for CD80/CD86 than CD28 and can reduce the expression of CD80/CD86 on the surface of DCs *via* trans-endocytosis. The co-expression of CD28 and CTLA-4 in Tregs, activated T cells, and their competitive combination with CD80/86 make the CD28/CTLA-4/B7 pathway play a more complex role in DC-Treg cell-mediated immune regulation (**Figure 2**).

The CD80/86-CD28 axis, the second signal-inducing Tregs produced by DCs, serves to lower the signaling threshold of the TCR, and induces the production of IL-2 that is essential for the development of CD4^+^ CD25^+^ Tregs. Besides, the CD80/86-CD28 axis also enables Treg cell homeostasis (7, 45, 46). Selectively targeting Tregs in normal Lewis rats with very low doses of the CD28 superagonist was shown to be sufficient to induce Treg cell proliferation *in vivo* and enable the effective treatment of EAE (47). Similarly, despite the normal number of Foxp3^+^ cells in Treg cell-specific CD28 conditional knockout mice, CD28-deficient Tregs exhibited a pronounced proliferative/survival disadvantage. This result caused a severe autoimmunity and experimental allergic encephalomyelitis that is considered as animal models of MS (48). However, it has been reported that CD28 knockout mice were resistant to EAE induction because the CD28 signals of pathogenic T cells and Tregs were blocked simultaneously (49). Additionally, selective blocking of CD80/86-CD28 signal transduction in rhesus monkeys suppressed the activation of autoreactive T cells and B cells, thereby abrogating the induction of EAE (50). Therefore, despite the pivotal role that the CD80/86-CD28 axis plays in the development and proliferation of tTregs and pTregs, it is evident that, the activation of the CD28 signal is conducive to the pathogenesis of EAE/MS, based on the overall perspective of the CD4^+^ T cell population. It’s probably because the CD80/86-CD28 axis has a much greater effect on the activation of pathogenic T cells than on the induction of Tregs, and pathogenic T cells have obvious quantitative advantages.

The CTLA-4-CD80/86 axis is an important medium for the Treg cell inhibition effect, which indirectly influences the activation of Teffs by suppressing the antigen-presenting function of DCs (51). Furthermore, CTLA-4 is an immune checkpoint and therapeutic target in the development of MS (52). For germline knockout mice, the deletion of CTLA-4 precipitates severe autoimmunity diseases (53). Real-time reverse transcription–PCR detection has shown that the expression of CTLA-4 in peripheral blood mononuclear cells (PBMCs) of MS patients was either decreased (54) or unchanged (55).These results, at least, could reflect the trend of CTLA-4 expression in Tregs of MS patients because CTLA-4 is mainly expressed by Tregs in PBMCs. In addition, a recent *in vitro* experiment demonstrated that when DCs were co-cultured with CD4^+^ CD25^−^ naive T cells and Tregs, Tregs always gathered around DCs, inhibited their activation, captured CD80/86 *via* trans-endocytosis of CTLA-4, and increased free PD-L1 expression on the surface of DCs (56, 57). This phenomenon influenced the antigen presentation ability of DCs, indirectly regulated the CD80/86-CD28 signaling pathway and inhibited the activation of T cells. Intriguingly, iTregs from CTLA-4-deficient mice failed to induce the downregulation of CD80/CD86, whereas DCs treated with iTregs from CTLA-4-deficient mice showed an impaired ability to activate naive T cells (57). Similarly, the CTLA-4-CD80/86 axis had the same effect on EAE mice, and the expression of CD80/86 and MHC-II on the surface of peripheral DCs was significantly elevated in Treg cell-specific CTLA-4-deficient mice (53). Thus, without the limiting effect of CTLA-4 on the Treg cell surface, the overall maturity and activation level of DC were enhanced. However, in the CNS of mouse, CTLA-4-deficient Tregs did not increase the expression of B7 on the surface of DCs (53). Moreover, in EAE/MS, Tregs can induce IDO expression in DCs *via* the CTLA-4-CD80/86 axis and improve their tolerance. Therefore, the CTLA-4-CD80/86 axis is of great significance in the DC-Treg cell crosstalk mechanism.

Although CTLA-4 is recognized as a co-inhibitory molecule, its effect on Tregs is highly complex. In general, it was thought that CTLA-4 promotes tTreg cell proliferation (51). For example, the lysine motif in the cytoplasmic domain of CTLA-4 was crucial for Foxp3 induction and EAE inhibition. Transporting the chimeric peptide containing the CTLA-4 cytoplasmic tail into T cells can induce Tregs *in vivo*, resulting in EAE remission (58). However, Paterson et al. (53) reported that CTLA-4 can limit the proliferation of Teffs and inhibit the expansion of pTregs, which is different from the role that CTLA-4 plays in the thymus. For example, a study showed that the deletion of CTLA-4 in adulthood promoted the activation and expansion of traditional CD4^+^ Foxp3^−^ T cell and regulatory Foxp3^+^ T cell subsets that protect mice from EAE (53). Paradoxically, Wing et al. (51) have suggested that CTLA-4 is necessary for Tregs to exert an inhibitory effect, whereas Paterson et al. (53) have proposed the opposite: upregulating the expression of other inhibitory molecules (e.g., IL-10, LAG-3, and PD-1) compensated for the CTLA-4 deficiency. There are two possible explanations for these results: 1. CTLA-4 expression has different relative contributions to Tregs and Teffs; and 2. the intracellular and extracellular segments of CTLA-4 are responsible for different functions.

Interference of the CD28/CTLA-4/B7 pathway can concurrently influence two signaling pathways between DCs and Tregs; therefore, its function in EAE/MS might be complicated. Abatacept is a CTLA-4-immunoglobulin (Ig) fusion protein, which is composed of the extracellular region of CTLA-4 and the Fc portion of IgG. It can simultaneously bind to CD80/86 and block the CD28/CTLA-4/B7 pathway. Glatigny et al. (59) found that abatacept significantly reduces circulating Tregs, especially CD45RO^+^ memory Tregs, in RRMS patients. Researchers suggested that the inhibition of CD28-mediated DC-Treg cell crosstalk was the primary mechanism underlying the reduction in human circulating Tregs following abatacept therapy (59). However, upon myelin reactive T cell activation, blocking B7 with CTLA-4-Ig can inhibit Tregs and aggravate EAE, resulting in more severe CNS inflammation and demyelination (60). It suggested that blocking the CD28/CTLA-4/B7 pathway primarily interferes with the CTLA-4-CD80/86 signal between DCs and Tregs, due to the reduced dependence of activated myelin-specific T cells on the CD28 signal. This interesting process caused Treg cell anergy and continuous activation of myelin-specific T cells, thus, promoting inflammation (60). In a phase II clinical trial called ACCLAIM (NCT01116427), abatacept provided no clinical benefit to patients with RRMS, which probably due to the mutual offset of the CD28 signal and the CTLA-4 downstream effect (61). Therefore, the final result of blocking the CD28/CTLA-4/B7 pathway in EAE/MS may depend on the relative strength of the CTLA-4 and CD28 signals as well as the blocking time point (61). However, no study to date has reported the effect of blocking the CD28/CTLA-4/B7 pathway in DCs in EAE/MS. Instead, studies in mouse models of rheumatoid arthritis have suggested that abatacept treatment induced a unique T cell phenotype to reduce DC antigen presentation (62).

In summary, in the context of MS/EAE, DCs rely on the CD80/CD86-CD28 axis to induce Treg cell generation, and Tregs rely on the CTLA-4-CD80/86 axis to inhibit DC immunogenicity, and induce the generation of tolDCs, thereby forming a protective signaling loop.

#### PD-L1/PD-1 inhibitory feedback loop

Similar to CTLA-4, the PD-L1/PD-1 axis also plays a pivotal role in inducing peripheral tolerance. However, the latter is distinct because it can be simultaneously expressed on the surface of DCs and Tregs (63, 64). Generally, cells with expression of PD-L1 play a regulatory role. PD-L1^+^ DCs involve in regulating the differentiation and development of naive T cells. Besides, they preferentially induce the expression of Foxp3 and promote the differentiation of Tregs. PD-L1^+^ DCs can also maintain Treg cell inhibitory function and promote the secretion of IL-10 by binding to PD-1 on the surface of Tregs (65). Simultaneously, PD-L1 can act inversely on DCs and inhibit DC maturation (6). In addition, PD-L1 constitutively expressed by Tregs can mediate the inhibitory function of Tregs, improve the tolerogenicity of PD-1^+^ DCs, and inhibit the secretion of proinflammatory cytokines, such as IL-6, IL-12 and tumor necrosis factor-alpha (TNF-α) (25, 66). Therefore, the PD-L1/PD-1 axis in DC-Treg cell crosstalk forms an inhibitory feedback loop and influences both cells simultaneously (**Figure 2**).

The PD-L1/PD-1 axis plays a similar protective role in MS. Numerous studies have reported the expression of PD-L1/PD-1 in MS patients or EAE mice from different perspectives, such as genes, peripheral blood, and brain tissue samples (66). Overall, the expression of PD-L1/PD-1 in healthy controls was highest, followed by MS patients or EAE mice in the remission stage, and finally those in the acute stage (66). However, exceptions also exist. For example, the expression level of PD-L1/PD-1 in MS plaques was higher than that in non-pathological human CNS tissue (67). Focusing on DCs/Tregs that are stimulated by MBPs, T cells expressing PD-1 and APC expressing PD-L1 in the peripheral blood of patients with acute MS were prominently increased compared with those of remitted patients with MS (68). A recent study also showed that the expression of PD-1 on the surface of CD4^+^ and CD8^+^ Tregs was reduced in patients with RRMS (69). Taken together, these studies suggested that the DC- or Treg cell-related PD-L1/PD-1 axis is involved in the occurrence and development of MS.

Tregs can improve DC tolerance *via* the PD-L1/PD-1 axis. As mentioned above, Tregs can release soluble PD-L1 on the surface of DCs when they endocytose CD80/86 in a CTLA-4-dependent manner, which boosts the tolerance of DCs (70). In addition, Liu et al. (25) found a new type of regulatory T cell subset: CD4^+^ FoxA1^+^ CD47^+^ CD69^+^ PD-L1^hi^ FoxP3^−^ Tregs (FoxA1^+^ Tregs). It was the main Treg cell subset that combats against EAE in the CNS and was induced by IFN-β. Transferring stable FoxA1^+^ Tregs to mice suppressed EAE in a PD-L1-dependent manner, which was related to its reduction in pro-inflammatory cytokines (IL-12 and IL-17) secreted by DCs and its inhibition of PD-1^+^ Teffs. Of note, FoxA1^+^ Tregs are negative for expression of Foxp3, CTLA-4, TGF-β, IL-10, and IL-35, though FoxA1 is a key transcription factor for PD-L1 production. Hence, the main pathway in which Tregs regulate DCs changes alongside alterations in the cell subtype. However, whether other Treg cell subtypes induce tolDCs *via* PD-L1 remains to be verified experimentally.

The regulation of PD-L1^+^ DCs on Treg proliferation, differentiation, and function in EAE/MS has attracted significant attention. A previous study in EAE has shown that steady-state DCs exhibited their suppressive capacity by promoting the development of Tregs, thereby improving CNS autoimmunity (6). Further research has demonstrated that the generation of DC-dependent Tregs was guided by the interaction between PD-L1 on DCs and PD-1 expressed by T Cells (6, 64). Paradoxically, one study showed that PD-L1 expressed on DCs was important for the inhibition of follicular regulatory T (Tfr) cell differentiation and maintenance (71). The study demonstrated that PD-L1 deficiency in DCs resulted in a higher percentage of Tfr cells in the draining lymph nodes and blood than in control mice. It can be preliminarily speculated that PD-L1^+^ CD11c^+^ DCs were responsible for inhibiting the development of CD4^+^ICOS^+^CXCR5^+^CD19^-^Foxp3^+^Tfr cells, but the specific DC subset was still unclear (71). We hold that this contradiction might be due to the heterogeneity of DC and Treg cell subsets. Moreover, PD-L1^+^ DCs have become a new therapeutic target for EAE/MS. Vitamin D3 (VitD3), IFN-β, hypomethylating agents, and other drugs can induce Tregs and alleviate EAE *via* PD-L1^+^ DCs (72, 73). A recent study showed that the IFN-γ-JAK1-STAT1 signaling pathway was an inherent upstream pathway that enabled DCs to induce PD-L1, followed by causing a series of subsequent immune tolerance effects. Therefore, mice harboring a DC-specific JAK1 deletion exhibited decreased expression of PD-L1 in DCs, fewer Tregs, and a deterioration of EAE (74). These studies demonstrated that the PD-L1/PD-1 axis played a powerful immune tolerance role in the DC-Treg cell crosstalk in the context of EAE/MS.

#### Potential LAG-3/TCR/MHC-II inhibitory feedback loop

The LAG-3 (CD223) is an inhibitory immune receptor that is mainly expressed by Tregs, especially Tr1, and negatively regulates T cell activation. Both TCR and LAG-3 on the surface of Tregs can bind to MHC-II on the surface of DCs. TCR-MHC-II signal transduction in DC-Treg cell crosstalk is the first signal produced by DCs inducing Tregs, and LAG-3-MHC-II signal transduction is one of the important ways for Tregs to inhibit DC antigen presentation. These two signal axes interfere with each other and form an inhibitory feedback loop (**Figure 2**).

Generally speaking, the TCR-MHC-II axis, as the first signal of T cell activation, plays a pathogenic role in EAE. However, it also plays a protective role in DC-Treg cell crosstalk. For instance, upon EAE induction, pDCs were recruited to the lymph nodes and established MHC-II-dependent cross-linking with CD4^+^ T cells to promote the selective amplification of myelin antigen-specific Tregs, thereby inhibiting the response of pathogenic T cells (75). DCs that express MHC-II at a low level facilitated a significant increase in the amount of nTregs and alleviated EAE (76). In turn, Tregs limited the antigen presentation ability of DCs by consuming the peptide-MHC class II complex on the surface of DCs. In addition, there was some evidence that LAG-3-MHC-II signal transduction in DC-Treg cell crosstalk had a protective effect in the context of autoimmunity disease. For example, basic studies and experiments on tumors had shown that LAG-3 competitively binded MHC-II with TCR, directly interfering with TCR signal transduction and blocking the DC-mediated immune response (77, 78). LAG-3 engagement with MHC-II inhibited DC activation and induced tolDC generation *via* the immunoreceptor tyrosine-based activation motif (ITAM)-mediated inhibitory signaling pathway that involved FcγRγ and the ERK-mediated recruitment of SHP-1 (78).

In addition, the expression level of LAG-3 is closely related to the course of MS. It has been reported that SNPs of LAG-3 influenced MS susceptibility (79). The expression of LAG-3 messenger RNA (mRNA) in PBMCs at diagnosis in untreated MS patients was significantly lower than that in healthy controls. Another study has shown that the reduced expression of baseline LAG-3 in PBMCs predicted the MS course alongside paraclinical and clinical parameters and might also be an adverse prognostic factor for MS (55). In line with this finding, a recent study observed significant reduction in the expression of LAG-3 mRNA in major CD4^+^ and CD8^+^ T cell subsets in RRMS individuals (80). Due to the expression level of LAG-3 in MS patients and the pathological characteristics of immune diseases, it can be speculated that the LAG-3/MHC-II axis is involved in DC-Treg cell crosstalk in MS/EAE and that the LAG-3/TCR/MHC-II inhibitory feedback loop mediates DC-Treg cell interactions and plays a protective role in MS/EAE.

### DC-Treg cell crosstalk-related inhibitory cytokines in MS/EAE

(**Figure 3**) Both DCs and Tregs can secrete IL-10/TGF-β/IL-35, and the secretion level is closely related to EAE/MS. It has been reported that the reduction in serum IL-10 level in patients with acute MS may be used as a marker of MS disease activity (81). As for cell subsets, the IL-10 secretion level of Tr1 is decreased in MS patients (82). In addition, increased TGF-β secretion in the peripheral blood of RRMS and SPMS patients has also been reported (83). Specifically, in MS patients, decreased gene expressions of key proteins in the TGF-β signal pathway of naive CD4^+^ T cells, such as TGF-βRI, TGF-βRII, and drosophila mothers against decapentaplegic 4 (SMAD4), have been reported (84), and T cell lines in patients with stable MS produce more TGF-β than those in patients with active MS (85). A recent study has further shown that a variety of cell surface markers related to TGF-β signal transduction in the Tregs are differentially expressed in MS patients and healthy controls, and are closely related to disease severity (86). These results indicate that TGF-β and its downstream signal pathway play a momentous modulating role in MS. Regarding IL-35, a new member of the IL-12 family, two contradictory studies have reported that the serum IL-35 level of RRMS patients was either lower or higher than that of healthy controls (87, 88). The subjects of the first study included both RRMS patients and clinically isolated syndrome (CIS) patients who have not yet converted to MS, which might be responsible for the contradiction of IL-35 expression level in serum. In addition, another study demonstrated that MS patients treated with IFN-β or methylprednisolone had higher serum IL-35 levels, which suggested that the clinical therapeutic benefits of these drugs might be partly induced by the upregulation of IL-35 (89). Collectively, MS patients can active the protective compensatory mechanism by regulating the secretion of IL-35 after symptoms onset, which may be influenced by the Foxp3 gene polymorphism and the progression stage of MS.

The above studies indicate that IL-10/TGF-β/IL-35 is involved in the regulation of MS disease activity and demonstrate their regulatory role in DC-Treg cell crosstalk. In addition, once cytokines have been secreted into the internal environment, it is difficult to determine their source and aim. For example, DC- or Treg- derived IL-10/TGF-β/IL-35 can influence the generation of Tregs by paracrine, but it also influences its own mature state and antigen presentation ability by autocrine. However, in order to clearly ascertain their roles in DC-Treg cell crosstalk, we distinguished them to DCs- and Tregs-derived cytokines in the following paragraphs.

#### DC-derived IL-10/TGF-β/IL-35 can promote Treg generation

DC-derived IL-10 can promote Treg cell generation and play a neuroprotective role (**Figure 3**). A recent study demonstrated that the adoptive transfer of tolDCs with upregulated IL-10 expression to EAE mice reduced the percentage of Th1 and Th17 cells, promoted splenetic Treg cell generation, and restricted the development of EAE to some degree (90). Besides, curcumin can promote the generation of peripheral IL-10-producing tolDCs and Tregs, as well as alleviate EAE, whereas the neuroprotection was completely abrogated in IL-10 deficient mice (91). In MS patients, there is a signal pathway of IL-10-dependent Treg cell generation: IL-10 activates signal transducer and activator of **t**ranscription 3 (STAT3) and converts DCs into tolDCs; in the presence of IL-2, tolDCs further induce STAT5 phosphorylation in naive T cells and promote Treg cell generation (92). Therefore, there is an inhibitory feedback loop about IL-10 that participates in DC-Treg cell crosstalk in MS patients. In addition, IL-10 is a downstream molecule of IL-27, and both of them established intravenous tolerance in EAE mice (93–95). For example, intravenously (i.v.) injecting MOG_35–55_ to wild-type (WT) mice infected with EAE can induce i.v. tolerance and reduce disease severity, whereas this administration was ineffective in IL-27 receptor (WSX-1) knockout mice. Moreover, research showed that distinct subsets of splenic DCs (i.e., CD11b^+^CD103^±^ and CD11b^hi^CD103^+^) contributed together to the induction of i.v. tolerance *via* promoting DC- drived IL-27, IL-10 and IFN-γ secretion, as well as suppressing IL-17 secretion (94).

DC-derived TGF-β promotes Treg cell generation *via* direct and indirect effects (**Figure 3**). It is well established that TGF-β is a popular inducer of Tregs *in vitro* that works independently of DC. In addition, several drugs alleviated EAE *via* the direct effect of TGF-β. For example, by targeting DCs to drive TGF-β1 upregulation, Tanshinone IIA(TSA) promoted the polarization of naïve CD4^+^ T cells to Tregs and significantly reduced the clinical severity of EAE (96). Apigenin shifted DC-modulated T cell responses from Th1 and Th17 types towards Treg cell-directed responses and curbed neuroinflammation in EAE by promoting the secretion of TGF-β and IL-10 (97). However, TGF-β can also promote the phosphorylation of IDO in DCs, a key enzyme in the tryptophan metabolic pathway, induce the DC tolerance phenotype, and indirectly facilitate Treg cell generation. For example, SMAD7 is an inhibitory SMAD protein that negatively regulates TGF-β signal pathway. Mice devoid of SMAD 7 in DCs were resistant to the induction of EAE. This result was predominantly caused by the high expression level of IDO and the elevated frequency of Tregs in secondary lymphoid organs and the CNS (98). Likewise, administration of the downstream tryptophan metabolite 3-hydroxyanthranillic acid enhanced the secretion of DC-derived TGF-β and the percentage of Tregs in IDO-deficient mice (99). Furthermore, the enhanced Treg cell response promoted the secretion of DC-derived TGF-β, formed an inhibitory feedback mechanism, and eventually reduced the clinical manifestations of EAE.

DC-derived IL-35 can promote Treg cell generation (**Figure 3**). IL-35-producing CD8α^+^ DCs have limited antigen presentation capacity and high tolerance. Moreover, since antigen-pulsed IL-35^+^ DCs possessed the ability to induce Tregs, their use can significantly reduce the severity of EAE, and Tregs induced by IL-35^+^ DCs acquired a stronger inhibitory function than those induced by TGF-β/IL-2 (100). Furthermore, the α (IL-12p35) and β (Epstein-Barr Virus-Induced, EBI3) subunits of IL-35 can both alter Tregs. For example, IL-12p35 suppressed neuroinflammatory responses and ameliorated the pathology of EAE by boosting the expansion of IL-10-producing Tregs in the brain and spinal cord as well as by inhibiting the STAT1/STAT3 pathways, and pathogenic Th17 and Th1 cells (101). Conversely, EBI3-deficient mice immunized by the MOG peptide elicited stronger Th17/Th1 responses in the CNS with marginally enhanced EAE development (102). Strikingly, there was an increased number of CD4^+^ Foxp3^+^ Tregs in the peripheral lymphoid organs of EBI3-deficient mice, and the suppressive function of these Tregs was more potent than Tregs in WT mice. The authors explained that the EAE development was only modestly enhanced compared with WT mice in the presence of potent Th17 response, which may attribute to the enhanced Treg responses (102). It’s indicated that the enhanced Treg responses was a comprehensive change of IL-35 and IL-27, because EBI3 is also the subunit of IL-27, which appears to have an inhibitory effect on Treg cell generation, as mentioned below. As a result, selectively knocking out IL-35-EBI3, while retaining IL-27-EBI3 may better reflect the effect of EBI3 subunit of IL-35 on Tregs.

#### Treg cell-derived IL-10/TGF-β/IL-35 can enhance the tolerance of DCs

Unfortunately, in the context of EAE/MS, there was little direct evidence for a distinct modulatory effect of Treg cell-derived IL-10/TGF-β/IL-35 on DC maturation and antigen presentation ability. Nevertheless, several basic experiments have shown that, in the context of immune tolerance, Treg cell-derived IL-10/TGF-β/IL-35 can acquire the capacity to inhibit DCs (**Figure 3**). For example, when Ag-specific iTregs were co-cultured with DCs in the absence of Teffs, iTreg cell-treated DCs had markedly impaired immunogenicity, which can be completely reversed by anti-IL-10 (57). Further research has shown that IL-10 can induce the degradation of CD86 and MHC-II on DCs and achieve the goal of suppressing the DC function by promoting the neutralization reaction between membrane associated ring-CH-type finger 1 (MARCH1, an E3 ubiquitin ligase) and CD83(a DC activation marker) (57). Another study reported that IL-10 can suppress immune responses by interfering with TLR- or IFNγ- mediated DC activation and directly inducing gene expression that inhibits APC function (103). Moreover, one study suggested that IL-10^-/-^ iTregs lost their protective role in EAE, which may be due to the impaired mechanism of iTreg cell-derived IL-10 inhibiting the expression of co-stimulatory molecules in DCs (104). A recent study demonstrated that Treg-derived IL-10 in tumors was also required for imprinting the Treg cell-inducing capacity of tolDCs (105).

Strikingly, a very recent report indicated that CD4^+^ Teff cell-derived IL-10 promoted CNS inflammation during the progression of EAE, *via* sustaining survival and proliferation of Th1 cells and, to a lesser degree, Th17 cells (106). However, IL-10 produced by Teffs did not exert its modulatory function by acting directly on APCs, and what affect such IL-10 may bear on the DCs remained open questions. Exploring the cellular source and targeted cell of cytokines seems to be important and meaningful. Besides, this study showed a hint: could Treg derived IL-10 also plays a pro-inflammatory role in a specific environment? This still needs further experiments and facts to prove. On the other hand, a study about gut inflammation also has reference value for MS because the microbiota-gut-brain axis is considered to participate in MS. For example, IL-10 signaling in CD11c^+^ DCs can limit reactivation of local memory T cells thereby controlling gut immune homeostasis (107). As mentioned above, the reactivation of memory T cells migrating to CNS is a pivotal process causing MS (3). Therefore, we speculated that IL-10 signal between CNS-DCs and memory T cells might be of similar significance. In conclusion, these researches proved the importance of exploring the cellular source and aims of cytokines, because IL-10 from different cellular sources may have different effects and mechanisms of action.

Treg cell-derived TGF-β is also necessary for the induction of DC tolerogenic function. When human monocytes were cultured with normal CD4^+^Foxp3^+^ Tregs and Th cells, they polarized into tolDCs, with capable of generating induced Tregs from naïve T cells. In this process, Treg-derived TGF-β, IL-10 and CTLA-4 were all required for the phenotypic differentiation of tolDCs (105). Similarly, a classical basic study revealed that Tregs restricted the maturation and antigen-presenting capacity of DCs, which can be marginally reversed using anti-TGF-β antibodies to neutralize TGF-β (108). Besides, transforming growth factor receptor 1 (TGFβR1) had an indispensable role in the development of intestinal CD103^+^CD11b^+^ DCs, and influenced the amount of DC related Foxp3^+^ Treg cells *in vitro* and *in vivo (* 109). These phenomena confirmed that Treg cell-derived TGF-β affected DC tolerogenic phenotype. As for IL-35, IL-35-treated MoDCs are characterized by tolerance, with a strong inhibitory function on the proliferation of pathogenic T cells. The exogenous addition of IL-35 inhibited MoDC maturation and secretion of pro-inflammatory cytokines, and also partially inhibited the differentiation of MoDCs (110). It suggested that Treg cell-derived IL-35 may also have a similar inhibitory effect on DCs.

In autoimmune diseases, Tregs inhibit DC maturation and improve immune tolerance by secreting IL-10/TGF-β/IL-35; moreover, DC provides conditions for Treg cell generation *via* the production of IL-10/TGF-β/IL-35. As a result, these three inhibitory cytokines involved in DC-Treg cell crosstalk formed three negative feedback mechanisms to counteract the imbalance of immune tolerance. Considering the autoimmune mechanism and the expression of IL-10/TGF-β/IL-35 in EAE/MS, we speculated that Treg cell-derived IL-10/TGF-β/IL-35 have a similar inhibitory effect on DCs in EAE/MS. However, it requires further experimental verification to fill the knowledge gap regarding cytokines in DC-Treg cell crosstalk. In addition, most researches have focused on the compherehensive changes in cytokines, whereas little attention has been paid to cytokine production in specific cell subsets. Therefore, unequivocally detecting cytokines is a critical future direction in the study of the DC-Treg cell crosstalk mechanism.

### Extracellular vesicles involved in DC-Treg cell crosstalk

Extracellular vesicles (EVs)—including exosomes that are wrapped with biological messengers, such as microRNAs and cytokines from DCs and Tregs—can complete cell interactions without direct contact (111, 112). Exosomes with membrane-associated TGF-β1 from gene-modified DCs can maintain the inhibitory function of Tregs, reduce the frequency of Th17 cells, and curb the progression of EAE (111, 112). Moreover, DC-derived exosomes can promote myelin regeneration (113). Conversely, with the help of EVs, Tregs can transport miRNA (especially mir-150-5p and mir-142-3p) into DCs to induce the tolerance phenotype (114). A previous study has shown that the inhibitory effect of Treg cell-derived exosomes in RRMS individuals was significantly weaker than that in healthy controls (115). Additionally, in a rat liver allograft model, exosomes from immature DCs were shown to retain numerous tolerogenic characteristics of immature DCs, and Tregs could be amplified in the presence of such exosomes to induce immune tolerance (116). Therefore, EVs are involved in DC-Treg cell crosstalk and are expected to become an emerging treatment strategy for MS.

### IDO1-related negative feedback loop

IDO is the first rate-limiting catabolic enzyme in the degradation pathway of the essential amino acid tryptophan (23). IDO has two isozymes: IDO1 and IDO2. IDO1 converts L-tryptophan to kynurenine *via* the kynurenine pathway (KP), which plays a key role in MS, whereas IDO2 may not be related to MS (117). Expressed mainly in DCs (especially pDCs), IDO typically has low basal expression, and its upregulation primarily correlates with cytokines (IFN-γ, IFNα/β, and TGF-β) and co-stimulatory signals (CTLA-4 and glucocorticoid-induced tumor necrosis factor receptor [GITR]) (118), whereas IL-6 and IL-4 inhibit the expression of IDO in DCs (119, 120). IDO activation is considered an endogenous self-protection response that accompanies EAE/MS (120). For example, the expression of IDO1 in the PBMCs of patients with RRMS was higher during the recurrence period than in the remission period (121). During an EAE attack in mice, activating the Stimulator of IFN Genes (STING) signaling adaptor to stimulate IFN-I production induced IDO expression in DCs, effectively inhibited disease progression, and alleviated clinical symptoms (122). Similarly, the mild condition of MS patients during pregnancy was related to the promotion of IDO expression in DCs by estriol. However, this compensatory effect is limited, and the IDO expression level in DCs of pregnant MS patients may be still lower than that of healthy controls (123, 124).

IDO1 mediates DC-Treg cell crosstalk and plays a protective role *via* the related negative signal loop (**Figure 3**). When EAE/MS occurs, both the massive secretion of IFN-α/β, IFN- γ, and CTLA-4-CD80/86 between Tregs and DCs can be used as the upstream signal of IDO1 to enhance the expression of IDO1 in DCs, which initiates tryptophan catabolism. Since tryptophan is an essential amino acid for T cell activation and proliferation, IDO1-mediated tryptophan catabolism can lead to tryptophan starvation, inhibit specific immune responses, and promote T cell anergy (8, 125). The KP is the most important tryptophan metabolism pathway mediated by IDO1. A variety of IDO1 downstream products produced *via* the KP are positive regulators that induce Tregs, of which kynurenine is the most representative. A large amount of kynurenine is produced and bound to AHR as a ligand, which promotes the generation of AHR-dependent Tregs (125). Moreover, such combination regulates the NF-kB pathway in DCs, further upregulates the gene expression level of IDO1, and improves the tolerance of DCs (119). In addition, long-term tryptophan catabolism is also conducive to promoting the differentiation of naive T cells into Tregs; and tolDCs can also enhance the inhibitory function of Tregs (119). Conversely, newly generated Tregs induce expression of IDO1 in DCs *via* CTLA-4, which further promotes the generation of tolDCs and forms the IDO1 related negative feedback loop. In addition, it has been demonstrated that in the steady state, Tregs induced the generation of IDO^+^ tolDCs in advance, and tolDCs, in turn, promoted the inhibitory function and rapid proliferation of Tregs following inflammation (8). Treg cell- or DC-derived TGF-β in EAE also induced IDO phosphorylation, promoted the gene expression level of TGF-β in DCs, and induced Treg cell generation. In the allergic airway inflammation model, Tregs induced IDO *via* GITR-GITR ligand (GITRL) reverse signal transduction (118). Surprisingly, basic research has also shown that IDO^+^ DCs can make IDO^-^ DCs tolerogenic *via* the bystander mechanism (126).

Compared with cDCs, pDCs have a stronger potential to induce Tregs in an IDO-dependent manner. However, the ability of pDCs to induce Tregs was significantly downregulated in MS patients, followed by destroying immune tolerance. Notably, IFN-β treatment can restore the Treg-inducing ability of pDCs (37). A newly developed DC-targeting IFN can specifically induce Tregs in an IDO-dependent manner to alleviate EAE (127). Its application eliminated the influence of IFN-β on other immune cells, which offered promise for providing an effective therapy. Furthermore, VitD3 and anti-malarial drug- primaquine, can also boost the expression level of IDO in CD11c^+^ DCs and induce Foxp3^+^ Tregs (128). The adoptive transfer of IDO^+^ CD11c^+^ DCs induced by VitD3 to EAE mice had the same therapeutic effect as oral or intraperitoneal injection of VitD3 (129). However, the role of IDO in EAE is variable. A recent study showed that the induction of EAE clinical signs was related to the activation of the neurotoxic metabolites of IDO and the KP, and the IDO inhibitor INCB024360 unexpectedly curbed the clinical symptoms and weight loss caused by EAE (120). The most likely explanation for this phenomenon is that IDO is a double-edged sword (130). In the early stage of inflammation, KP maintained immune tolerance by promoting the generation of Foxp3^+^ Tregs and played a protective role in EAE. However, as the disease progresses, the infiltration of immune cells in the CNS intensified, and microglia produced neurotoxic metabolites of kynurenine, such as quinolinic acid (QUIN) and 3-hydroxykynurenine, downstream of IDO. These toxic substances accumulated in the prefrontal cortex, hippocampus, spinal cord, spleen, and lymph node of EAE mice, gradually offsetting the IDO-induced immune tolerance (120). Among these metabolites, QUIN connected neuroinflammation with neurodegeneration and guided EAE to develop in the direction of neurodegeneration-related chronic inflammation and neurotoxicity (120, 131). Therefore, IDO inhibitors are expected to become a new strategy for the treatment of advanced MS with neurodegenerative diseases; selecting the right time point to administer IDO inhibitors and treat autoimmune diseases will be a challenge in the future.

## Unidirectional regulation of Tregs by DCs in MS/EAE

Various signal loops with surface molecules, cytokines, and metabolic enzymes as the core, as described above, explain the majority of the DC-Treg cell crosstalk mechanism; however, there are also other molecules, such as thymic stromal lymphopoietin (TSLP), GITRL, IL-27, and retinoic acid (RA), indirectly influence Tregs by inducing DC tolerogenicity. Unsatisfactorily, it has not been confirmed experimentally whether Tregs participate in the regulation of DC through these molecules.

### Surface molecules involved in the DC regulation of Tregs

DCs can regulate Treg cell generation with the help of surface molecules (**Figure 2**). For example, TSLP-activated DCs can provide a strong survival signal for high-affinity autoreactive T cells and actively participate in their differentiation into Tregs (132). The expression level of the TSLP receptor (TSLPR) on the surface of myeloid DCs was decreased in peripheral blood of MS patients, and the function of DCs was altered, which resulted in abnormal Treg cell homeostasis and function. Upon immunomodulatory treatment with IFN-β and glatiramer acetate, TSLPRs on myeloid DCs were upregulated, and Treg cell homeostasis was restored (133). These studies demonstrated that TSLPRs were involved in the generation of DC-dependent Tregs.

The binding of GITRL on the DC surface and the corresponding receptor (i.e., GITR) on the Treg cell surface can improve the sensitivity of Treg precursor cells to IL-2 and provide co-stimulatory signals for Treg cell proliferation (134, 135). A recent study showed that the expression of GITRs on the surface of CD4^+^ and CD8^+^ Tregs was decreased in RRMS individuals (69). This might be one of the reasons that DC-Treg cell crosstalk is destroyed in MS patients, which limits the proliferation of DC-dependent Tregs. In addition, the GITR-GITRL axis was one of the mechanisms underlying the treatment effect of IFN-β on MS. It has been shown that in the presence of IL-2, IFN-β induced the expression of GITRL on DCs, enhanced the GITR-GITRL axis signal transduction, promoted the proliferation of Tregs, and participated in the treatment of MS (136). The allergic airway inflammation model suggested that the reverse signal transduction of GITRL induced IDO expression in mouse pDCs (118). Thus, the GITR-GITRL axis might also play a bidirectional role in DC-Treg cell crosstalk in EAE/MS.

CD31 is a transhomophilic tyrosine-based inhibitory motif receptor expressed by DCs. Sustaining CD31/SHP-1 signaling during DC maturation resulted in reduced NF-κB nuclear translocation and enhanced tolerogenic functions of DCs that promoted the generation of antigen-specific Tregs (137). The adoptive transfer of CD31-conditioned MOG-loaded DCs can significantly increase the percentage of CD4^+^ CD25^+^ Foxp3^+^ Tregs and offer immune tolerance against the subsequent development of MOG-induced EAE *in vivo* (137). Therefore, inducing Treg cell generation by regulating these DC surface molecules is a potentially promising treatment for MS.

### Cytokines involved in the DC regulation of Tregs

#### DC-derived IL-27 induces Treg cell generation

IL-27 secreted by DCs has both anti-inflammatory and pro-inflammatory effects, and its production changes with the occurrence of MS/EAE (138). For example, plasma IL-27 secretion and IL-27 mRNA expression in PBMCs were significantly decreased in patients with progressive MS (139). The IL-27 subunit and its receptor were upregulated in the CNS inflammatory cells of EAE mice (140). These results suggested a clear correlation between IL-27 and EAE/MS.

Studies have shown that the IL-27 signaling pathway mediates protective DC-Treg cell crosstalk in EAE (141) (**Figure 3**). For example, IL-27 receptor knockout mice with EAE deteriorate significantly, accompanied by reductions in the frequency of Foxp3^+^ and IL-10^+^ CD4^+^ T cells (141). Subsequent studies have shown that DC-derived IL-27 activated STAT1, STAT3, AHR, c-Maf, and other transcription factors, induced the production of IL-10, and, in turn, promoted the generation of Tr1 and Tregs (22, 93). Recent studies have revealed that the functioning way of IL-27 differed between the central and peripheral tolerance. Do et al. (142) used a Treg cell-specific IL-27 receptor knockout mouse model to illustrate that IL-27 signaling in Foxp3^+^ Tregs was essential for Tregs to control autoimmune inflammation in the CNS. However, Thomé et al. (94) demonstrated that this classical IL-27 signaling mode in T cells was not necessary to induce peripheral tolerance in EAE mice. In contrast, the indirect way in which IL-27 promotes DC tolerance and induces Treg cell generation is more pivotal in peripheral tolerance. In addition to secreting IL-10, IL-27 plays an anti-inflammatory role in another two ways to induce tolDCs and prevent the development of CNS inflammation and EAE: 1) promote the expression of co-inhibitory molecules PD-L1 and CD39 on the surface of DCs (22, 141, 143, 144); 2) regulate the NF-κB signaling pathway in DCs to promote the expression of TGF-β1 and IDO (137).

IL-27 appears to have a dual effect on Treg cell generation in EAE mice (93, 145). For example, although the systemic delivery of IL-27 can effectively prevent the initiation of Th17 cells and the development of EAE, it enhanced the Th1 response, downregulated the frequency of Foxp3^+^ Tregs in the spleen, and significantly inhibited Treg cell subsets of the CNS without influencing Tr1 cells (146). This phenomenon increased the risk of IL-27-based EAE treatment and suggested that IL-27 has an inhibitory effect on Treg cell generation. In addition, IL-27 in EAE also inhibits immune tolerance mediated by mature DCs and thus plays a pro-inflammatory role. In this process, mature DCs treated with IL-27 did not influence the expression of Treg cell-associated molecules on CD4^+^ T cells *in vivo* or *in vitro* but induced the development of CD4^+^ CD127^+^ 3G11^+^ Tregs, which were considered an unprecedented pro-inflammatory Treg cell subset (138). Therefore, the mechanism of the pleiotropic molecule IL-27 in DC-Treg cell crosstalk has not been fully elucidated. At present, in the context of EAE/MS, most studies are devoted to the anti-inflammatory effect of IL-27 on the promotion of the generation of tolDCs and Tregs. However, the pro-inflammatory effect of IL-27 and its negative regulation of Treg cell generation also warrant further attention.

#### Other cytokines that induce Tregs in a DC-dependent manner

RA is produced by a variety of cells, including DCs and Tregs (**Figure 3**). Although numerous studies have shown that RA directly promotes Treg cell proliferation (147, 148), there is evidence that RA can also indirectly influence Tregs in a DC-dependent manner. For example, in an EAE model, all-trans-RA can impair the antigen-presenting ability of DCs, resulting in the reduction of pro-inflammatory Th1/Th17 cells and severity of EAE (149). Similarly, helminth-infected MS patients developed a relatively mild condition owing to the increased synthesis of RA in DCs, so that DCs were programmed to develop into tolDCs, which induce Foxp3^+^ Tregs and inhibit the production of the suppressor of cytokine signaling 3-mediated pro-inflammatory cytokines (150). Furthermore, tolDCs induced by RA can induce the antigen-specific Treg cell response *in vitro*, and the liposomal co-delivery of antigen and RA might be a more targeted approach to induce antigen-specific tolerance in autoimmune and chronic inflammatory diseases (148). Thus, RA is of great significance for DC-dependent Treg cell generation.

Most galectins are critical mediators for the induction of peripheral tolerance, offering protective effects for MS patients and EAE mice. Galectin-1 is synthesized and secreted by Tregs, DCs, and other cells, which in turn boosts the generation of tolDCs. A previous study showed that galectin-1 induced tolDCs, enhanced the development of Tr1 and Tregs, and participated in the induction of intravenous tolerance in MOG_35-55_ immunized EAE mice (151). In addition, EAE was exacerbated in Lgals8/Lac-Z knock-in mice lacking galectin-8 expression because of the increased immunogenicity of DCs and the disruption of the Treg cell-Th1/Th17 cell balance (152).

### Possible pathways of tolDCs inducing Tregs

The contribution of the immunoglobulin-like transcript (ILT), CD83, and heme oxygenase 1 (HO-1) to DC-Treg cell crosstalk in MS/EAE has not been extensively reported. However, researches suggested that they indirectly participate in the DC-Treg cell interplay by inducing tolDCs.

The inhibitory receptors ILT3 and ILT4 are vital for inducing T cell tolerance, highly expressed in tolDCs, and independently prevent the activation of pathogenic T cells (14). The beneficial effect of IFN-β in MS patients was probably achieved partially by regulating the expression of ILT3 and ILT4 in DCs. VitD3 also increased the expression of ILT3 but not ILT4 (153). These studies suggested that a variety of drugs induce Tregs and treat MS by regulating the expression of ILTs on the surface of DCs. Besides, *via* secreting IFN-I, toll-like receptor 9-activated cDCs can regulate neutrophil and monocyte trafficking to the inflamed colon and restrain the inflammatory products of tissue macrophages, thereby inhibiting colonic inflammation independently of T cell (154). This reminds us that cDCs may further influence CNS inflammation in a similar way, with the help of gut-brain axis. In addition, CD83 is expressed in activated B cells, T cells, and especially Tregs, and its soluble form inhibits the maturation and function of DCs (155). CD83 transgenic mice recovered quickly from EAE with the enhanced activity of Tregs and the limited proliferation of pathogenic T cells (155). Therefore, CD83 probably induced Treg cell generation in a tolDC-dependent manner.

Surprisingly, numerous studies have shown that HO-1 has a protective effect on MS/EAE. HO-1 in EAE inhibited the expression of MHC-II in DCs and improved DC tolerogenecity (156–158). However, one study found that HO-1 did not regulate the number or function of infiltrated Tregs in the CNS; it showed that the protective effect of HO-1 on the CNS of EAE mice might not be related to DC-Treg cell crosstalk (159). Currently, it remains uncertain whether peripheral HO-1^+^ DCs in EAE play a protective role *via* Tregs, because in the airway inflammation model, HO-1 expressed by DCs indeed promote the differentiation of Tregs (159).

## Potential tolDC subsets can induce Tregs

There are a small number of dedicated so-called tolerogenic DC subsets, which are predestined to induce Tregs in a steady state (**Table 3**). Moreover, the generation of tolDC is closely related to the Wnt/beta-catenin pathway. A classical study in 2007 demonstrated that disruption of E-cadherin-mediated adhesion could induce peripheral T cell tolerance *in vivo* and protect against EAE (164). In this process, E-cadherin triggered Wnt/beta-catenin pathway, a classical pathway to induce DC tolerance and maturation, and these tolDCs further boosted IL-10-producing Treg cell generation and peripheral tolerance (164, 165). Of note, CD103 is a heterotypic ligand for E-cadherin, so some CD103^+^DCs might be a potential tolDC subset. For example, Langerin^+^CD103^+^ migratory DCs had a superior ability to generate Tregs *in vivo*, which in turn drastically alleviated EAE (160). Besides, a recent study defined another tolDC subset: BTLA^+^ DEC205^+^ CD8^+^ CD11c^+^ DCs, which took advantage of the engagement of the herpesvirus entry mediator (HVEM), a receptor of BTLA, upregulated the expression of Foxp3 in a CD5-dependent manner, and promoted peripheral Treg cell transformation (161). This study suggested that the cross-linking of BTLA and HVEM was one of the key mechanisms for DC to induce peripheral Tregs in EAE. However, most CD11c^+^ DCs are BTLA^neg^ DCs and thus can’t acquire tolerance even in a steady state. Moreover, IL-10-producing DC(DC-10) is also a classical tolDC subset, and most of the induced tolDCs in EAE/MS are recognized by the secretion of IL-10. Therefore, we deem that DC-10 is a potential tolDC subset, but its concept is not clearly proposed in EAE (162, 163). Moreover, it should be noted that tolDC subsets only exist in the steady state. Once inflammation occurs, tolDCs would play a pro-inflammatory role again and activate Teffs. At that time, induced-tolDC would be crucial in role of immune protection.

**Table 3:** Potential tolDC subsets that can induce Treg cells.

TolDC phenotype	Key molecules	Relevant Treg cell phenotype	Distribution	Ref.
Langerin^+^ CD103^+^ migratory DC	Langerin and CD103	Foxp3^+^Treg	Peripheral tissues	(160)
Interleukin-27-producing CD11b^+^CD103^±^/ CD11b^hi^CD103^+^DC	IL-27	Foxp3^+^Treg and Tr1 cell	Spleen	(94)
BTLA^+^DEC205^+^CD8^+^CD11c^+^ DC	BTLA	CD4^+^CD25^+^Foxp3^+^Treg	Lymph nodes and spleen	(161)
Interleukin-35-producing CD8α^+^ DC	IL-35	IL-35^+^ DC-regulated Tregs do not express Foxp3, but their suppressive capacity is more potent than TGF-β /IL-2-induced Tregs.	/	(100)
CD31^+^ DC	CD31	CD4^+^ CD25^+^ Foxp3^+^ Treg	Draining lymph nodes	(137)
DC-10: CD1a^-^CD1c^-^CD14^+^ CD16^+^ CD11c^+^CD11b^+^HLA-DR^+^CD83^+^ IL-10-producing DC (human) (include two subsets: CD83^high^ CCR7^+^ DC and CD83^low^CCR7^-^ DC)	IL-10	IL-10-producing Tr1 cell	Peripheral blood and secondary lymphoid organs	(162, 163)

## DC-Treg crosstalk mechanism can be applied to MS treatment

DC-Treg crosstalk is one of the pivotal mechanisms of drugs to alleviate MS, such as IFN-β, Vit D3, tanshinone IIA, primaquine and apigenin. Besides, as for DC-Treg crosstalk, the treatment of MS/EAE in the future can also be explored in the following aspects: 1) inhibitors, agonists, or specific antibodies of critical immune targets, e.g., DC-targeting CTLA-4-Ig and PD-L1/PD-1 agonists; 2) therapeutic interventions modulating IDO activity, e.g., IDO inducers/inhibitors and STING agonists. However, drug-based therapy mostly plays its role through non-specific immunity, which often leads to side effects such as decreased immunity and infection. Therefore, cellar-based therapy involving tolDCs and Tregs have become a new upsurge, such as peptide- and protein-based vaccines. It can reestablish antigen-specific immune tolerance towards CNS structures and control autoreactive T cells without inducing systemic immune suppression.

At present, peptide-based vaccines were advanced into human clinical trials. A phase I clinical trial (NCT02283671) starting at 2014, was for the first time to evaluate the tolerance and safety of tolDCs in treating patients with MS or neuromyelitis optica (NMO). The clinical use of tolDCs in a well-defined population of MS patients was evaluated in two subsequent phase I clinical trials (NCT02618902 and NCT02903537). These two clinical trials conducted dose-escalation studies and compared each other through intradermal and intranodal cell administration, followed by evaluating the safety and feasibility of tolDC administration, and exploring appropriate vaccine injection doses and DC delivery methods for the next phase II clinical trials (166). In addition, a phase II clinical trial (NCT04530318) about the treatment of MS with autologous peripheral blood differentiated tolDCs combined with immune regulation was started in 2020, which is expected to be completed in 2024. Surprisingly, the current experiment did not report any safety problems related to tolDC administration in MS patients (166).

Similarly, protein-based vaccines are also intriguing tolDC therapies that involve DC-specific antigen-targeting strategy. For instance, both DEC205 (CD205) and dendritic-cell inhibitory receptor-2 (DCIR2), are DC-specific endocytosis receptors, which can improve the antigen presentation efficiency of DCs (167, 168) (**Figure 2**). Delivering PLP to DCs using αDEC-205 fusion monoclonal antibodies or DCIR2 fusion monoclonal antibodies can induce Treg cell generation. Then these Tregs resulted in antigen-specific tolerance and amelioration of EAE (167, 169). Similarly, targeting migratory DCs with an anti-receptor-antigen fusing to MOG can provide the same result (160). Intriguingly, αDEC-205^+^ fusion antibodies cause extrathymic induction of a Foxp3^+^ Treg cell phenotype in naïve CD4^+^ Foxp3^−^ T cells, whereas DCIR2^+^ fusion antibodies result in the proliferative expansion of natural Foxp3^+^ Tregs in EAE (167). Besides, many drugs are inducers of tolDCs, such as interferon, vitD3, statins, galectin-1, tofacitinib, etc (151, 170, 171). Combining these drugs with protein-based vaccines is also more conducive to the induction of tolDCs.

## Conclusion

DC-Treg cell crosstalk involves a variety of surface molecules, cytokines, and metabolic enzymes *via* multiple inhibitory feedback loops and plays a key protective role in the silent progression of MS. Drugs influencing DC-Treg crosstalk and tolDC therapy are expected to emerge as promising treatment strategies for MS. However, selecting the appropriate time point and specifically targeting DCs/Tregs without affecting the immune state of other cells is worthy of future discussion, due to different onset times and the wide distribution/rich targets of these immune effector molecules. At present, the researches of functional molecules working comprehensively become more and more popular in the environment of CSF, PBMC or peripheral blood in MS patients. Although, these studies can well show the correlation between molecules and diseases, what unsatisfied was that they did not reflect the molecular changes in specific cell subtypes and we cannot distinguish the cellular source and targeted cells of functional molecules from those studies. Therefore, further research on the expression level of molecules based on cell subsets is conducive to a more comprehensive analysis and evaluation of molecular effects. In addition, the increased focusing on tolDC therapy has facilitated an upsurge in research on DC-induced Tregs, whereas investigations on the effect of Tregs in DCs remain relatively scarce. There remain several knowledge gaps in the mechanism of DC-Treg cell crosstalk, and further research would be necessary to translate mechanisms into clinical therapy.

## Author contributions

RL, PL, and HLiu conceived this review. RL performed specific database queries, generated the figures and tables, and wrote the manuscript. HLi, XY, HH, PL, and HLiu edited and revised the review. All authors contributed to the article and approved the submitted version.

## Funding

This work was supported by Henan Provincial and Ministerial Co-construction Projects (No. SB201901018) and the National Natural Science Foundation of China (No. U2004128).

## Acknowledgments

We thank the reviewers for their insightful and constructive comments on the manuscript. We are grateful to the translational medicine center of the First Affiliated Hospital of Zhengzhou University for support.

## Conflict of interest

The authors declare that the research was conducted in the absence of any commercial or financial relationships that could be construed as a potential conflict of interest.

## Publisher’s note

All claims expressed in this article are solely those of the authors and do not necessarily represent those of their affiliated organizations, or those of the publisher, the editors and the reviewers. Any product that may be evaluated in this article, or claim that may be made by its manufacturer, is not guaranteed or endorsed by the publisher.
